# Draft Genome Sequences of the 1,2-Dichloropropane-Respiring Dehalococcoides mccartyi Strains RC and KS

**DOI:** 10.1128/MRA.01081-18

**Published:** 2018-09-13

**Authors:** Steven A. Higgins, Elizabeth Padilla-Crespo, Frank E. Löffler

**Affiliations:** a Department of Microbiology, University of Tennessee, Knoxville, Tennessee, USA; b Department of Civil and Environmental Engineering, University of Tennessee, Knoxville, Tennessee, USA; c Department of Biosystems Engineering and Soil Science, University of Tennessee, Knoxville, Tennessee, USA; d Center for Environmental Biotechnology, University of Tennessee, Knoxville, Tennessee, USA; e Biosciences Division, Oak Ridge National Laboratory, Oak Ridge, Tennessee, USA; Queens College

## Abstract

Dehalococcoides mccartyi strains RC and KS respire toxic 1,2-dichloropropane to environmentally benign propene. Their genomes were sequenced with Ion Torrent technology, assembled, and annotated. The draft genomes of strains RC and KS were 1.50 and 1.49 Mb in size and carried 1,653 and 1,671 genes, respectively.

## Abstract (precis)

Dehalococcoides mccartyi strains RC and KS respire toxic 1,2-dichloropropane to environmentally benign propene. Their genomes were sequenced with Ion Torrent technology, assembled, and annotated.

## ANNOUNCEMENT

Dehalococcoides mccartyi strains RC and KS conserve energy and produce innocuous propene and inorganic chloride from the dichloroelimination of 1,2-dichloropropane (1,2-D), a chlorinated aliphatic hydrocarbon regulated to less than 5 ppb in groundwater (1). The 1,2-D dechlorinating D. mccartyi strains RC and KS possess a 1,2-D reductive dehalogenase (RDase) gene, *dcpA* (2), yet share >99% 16S rRNA gene sequence identity with D. mccartyi strains that cannot grow on 1,2-D. To support comparative genome and trait analyses of D. mccartyi strains, we prepared draft genome sequences of strains RC and KS, which were identified as the key 1,2-D dechlorinators in enrichment cultures comprising strains RC and KS, respectively.

Cultures of RC and KS were derived from sediment samples collected from the Red Cedar River in Okemos, Michigan, and the King Salmon River in Alaska, respectively (3). Antibiotic treatment and continuous transfers to completely synthetic, defined mineral salts medium amended with 1,2-D as the electron acceptor and hydrogen as the electron donor resulted in highly enriched bacterial communities dominated by D. mccartyi (1–3). DNA was extracted from 10^9^ D. mccartyi cells by using a PowerWater kit (Mo Bio, Qiagen, Venlo, Netherlands) and treated with RNase I (Sigma-Aldrich, St. Louis, MO, USA). Sequencing libraries were prepared using an Ion Xpress Plus fragment library kit and an Ion Xpress Template 200 kit (Thermo Fisher Scientific, Waltham, MA, USA) and sequenced using an Ion 316 chip kit on the Ion Torrent PGM sequencer (1 × 200-bp libraries). A total of 3,640,282 and 4,501,960 reads passed quality control (average Phred score, ≥20; minimum read length, 50 nucleotides [nt]) with average read lengths of 148.0 ± 79.4 and 176.4 ± 65.2 nt for strains KS and RC, respectively. A random subsample of 600,000 reads from each consortium metagenome was assembled using Newbler v2.6 (default settings). Totals of 45 and 23 contigs were generated for strains RC and KS, respectively. Contigs failing to align to genomes of representative *Dehalococcoidia* strains were excluded, and remaining contigs were scaffolded with SIS software (default settings) (4) using the genome of D. mccartyi strain DCMB5 as a reference (99.0 and 99.6% genomic average nucleotide identity against strains RC and KS, respectively). The final draft genomes of strains RC and KS were assembled into 25 and 23 contigs totaling 1,502,842 and 1,485,739 bp, with *N*_50_ values of 210,101 and 268,024 bp and G+C contents of 47.17% and 47.23%, respectively.

Genome annotation using the RAST server (5) identified 1,653 and 1,671 gene sequences for strains RC and KS, respectively. A total of 46 tRNA genes were detected, along with single copies of the 5S, 16S, and 23S rRNA genes, which shared 99 to 100% nucleotide identity to rRNA genes in other D. mccartyi strains. The 16S rRNA genes were detected within a distinct contig separate from the colocated 23S and 5S rRNA genes. The draft genomes of strains RC and KS contained 38 and 31 putative RDase genes, respectively, 25 of which possessed cognate RDase B genes. With the exception of *dcpA*, all RDase genes were determined to be orthologs of known D. mccartyi genes, including *pcbA4*, implicated in reductive dechlorination of polychlorinated biphenyls (6). The genomes of strains RC and KS expand the sequence space of organohalide-respiring *Chloroflexi* spp. and knowledge of the genetics underlying 1,2-D dechlorination.

### Data availability.

This whole-genome shotgun project has been deposited in DDBJ/ENA/GenBank under the accession numbers QGLD00000000 and QGLC00000000. The versions described in this paper are versions QGLD01000000 and QGLC01000000. The raw sequence data are available within the Sequence Read Archive under the accession numbers SRR7653600 and SRR7653601.

## References

[1] Löffler FE, Champine JE, Ritalahti KM, Sprague SJ, Tiedje JM 1997 Complete reductive dechlorination of 1,2-dichloropropane by anaerobic bacteria. Appl Environ Microbiol 63:2870–2875. 16535654 10.1128/aem.63.7.2870-2875.1997 PMC1389209

[2] Padilla-Crespo E, Yan J, Swift C, Wagner DD, Chourey K, Hettich RL, Ritalahti KM, Löffler FE 2014 Identification and environmental distribution of *dcpA*, which encodes the reductive dehalogenase catalyzing the dichloroelimination of 1,2-dichloropropane to propene in organohalide-respiring *Chloroflexi*. Appl Environ Microbiol 80:808–818. doi:10.1128/AEM.02927-13. 24242248 PMC3911209

[3] Ritalahti KM, Löffler FE 2004 Populations implicated in anaerobic reductive dechlorination of 1,2-dichloropropane in highly enriched bacterial communities. Appl Environ Microbiol 70:4088–4095. doi:10.1128/AEM.70.7.4088-4095.2004. 15240287 PMC444787

[4] Dias Z, Dias U, Setubal JC 2012 SIS: a program to generate draft genome sequence scaffolds for prokaryotes. BMC Bioinformatics 13:96. doi:10.1186/1471-2105-13-96. 22583530 PMC3674793

[5] Aziz RK, Bartels D, Best AA, DeJongh M, Disz T, Edwards RA, Formsma K, Gerdes S, Glass EM, Kubal M, Meyer F, Olsen GJ, Olson R, Osterman AL, Overbeek RA, McNeil LK, Paarmann D, Paczian T, Parrello B, Pusch GD, Reich C, Stevens R, Vassieva O, Vonstein V, Wilke A, Zagnitko O 2008 The RAST server: Rapid Annotations using Subsystems Technology. BMC Genomics 9:75. doi:10.1186/1471-2164-9-75. 18261238 PMC2265698

[6] Wang S, Chng KR, Wilm A, Zhao S, Yang K-L, Nagarajan N, He J 2014 Genomic characterization of three unique *Dehalococcoides* that respire on persistent polychlorinated biphenyls. Proc Natl Acad Sci U S A 111:12103–12108. doi:10.1073/pnas.1404845111. 25028492 PMC4142991

